# Stress-associated testosterone suppression: central adaptation or hypogonadism?

**DOI:** 10.1210/clinem/dgag181

**Published:** 2026-04-27

**Authors:** Karl E Friedl, Bradley C Nindl, Adam W Potter

**Affiliations:** U.S. Army Research Institute of Environmental Medicine, Natick, MA 01760-5007, USA; Neuromuscular Research Laboratory, University of Pittsburgh, Pittsburgh, PA 15203, USA; U.S. Army Research Institute of Environmental Medicine, Natick, MA 01760-5007, USA

**Keywords:** testosterone, hypothalamic–pituitary–gonadal axis, energy availability, sleep restriction, military training, stress, sex hormone–binding globulin

## Abstract

Low circulating testosterone in physically stressed populations is frequently interpreted as evidence of hypogonadism or intrinsic gonadal dysfunction. However, convergent data from military field studies, endurance athletes, and competitive stress models demonstrate that testosterone suppression during sustained stress is commonly a centrally mediated, reversible adaptation rather than intrinsic testicular failure. Severe energy deficit, sleep disruption, and uncontrollable psychogenic stress suppress hypothalamic gonadotropin-releasing hormone and luteinizing hormone pulsatility, reduce testicular androgen production, and frequently increase sex hormone–binding globulin (SHBG), thereby disproportionately lowering free testosterone. Human chorionic gonadotropin stimulation studies confirm preserved Leydig cell responsiveness under these conditions, supporting hypothalamic–pituitary inhibition as the dominant mechanism. In contrast, high mechanical loading in resistance-trained men does not suppress basal testosterone when energy availability is maintained, underscoring energetic sufficiency, not exercise modality, as the principal determinant of androgen tone. Acute competitive stress produces rapid, appraisal-dependent modulation of testosterone independent of SHBG, further demonstrating central regulation. Across contexts, androgen suppression tracks energetic and psychological constraint and is reversible with restoration of energy balance and recovery. Recognition of this adaptive endocrine phenotype is essential to distinguish functional central suppression from pathological hypogonadism and guide appropriate clinical evaluation.

Testosterone concentrations decline in a wide range of physiological stress states, including sustained military operations, endurance training under energy deficit, sleep restriction, and severe psychological strain (1-5). These reductions are frequently interpreted as evidence of hypogonadism, impaired gonadal function, or diminished health (6, 7). Previous reviews have summarized clinical aspects of male hypogonadism in the context of systemic illness and aging. The present review extends this literature by integrating historical military field investigations, controlled multistressor experiments, and athletic models to demonstrate that testosterone suppression under sustained stress represents a coordinated hypothalamic adaptation rather than intrinsic gonadal dysfunction (2, 8, 9).

Reproductive function is energetically costly (10, 11). Androgen-dependent processes, including spermatogenesis, erythropoiesis, and maintenance of metabolically active lean tissue, require substantial substrate availability and neuroendocrine support (12-14). Under conditions of sustained energetic constraint or uncontrollable stress, central regulatory systems reprioritize physiological functions toward immediate survival, immune defense, and cognitive performance (15, 16). Suppression of gonadotropin-releasing hormone and luteinizing hormone pulsatility reduces testicular androgen production (8, 9), but increases in sex hormone–binding globulin may then further lower a free testosterone component (3, 17). However, because free testosterone is typically calculated rather than directly measured, its interpretation depends on assumptions regarding binding equilibria and SHBG concentrations. Consequently, calculated values should be interpreted alongside total testosterone and SHBG rather than as independent analytical variables.

Acute stress exposure may transiently stimulate or leave luteinizing hormone (LH) secretion unchanged in short-duration contexts (18). In contrast, sustained energetic and psychological strain consistently suppresses LH pulse amplitude and frequency, establishing centrally mediated suppression of gonadotropin drive as the defining feature of prolonged stress-associated androgen reduction. Human stimulation studies demonstrate preserved Leydig cell responsiveness during these states, indicating intact gonadal capacity despite reduced circulating concentrations (1, 9, 19).

Distinguishing functional central suppression from intrinsic hypogonadal pathology has significant clinical implications. Failure to account for energy availability, sleep integrity, psychogenic stress exposure, high physical workload, and body composition may lead to misclassification of reversible physiological adaptation as endocrine disease (3, 20-22). This review synthesizes evidence from military field investigations, controlled training studies, athletic models, and competitive stress paradigms to define the mechanisms governing testosterone regulation under sustained stress and to propose a framework for clinical interpretation grounded in hypothalamic–pituitary physiology rather than isolated serum measurements.

## Methods

Literature cited in this narrative review was identified through searches of PubMed and Web of Science using combinations of the terms *testosterone*, *stress*, *military training*, *energy deficiency*, *sleep restriction*, *HPG axis*, and *exercise*. Priority was given to controlled human experiments, military field investigations, and mechanistic studies of hypothalamic–pituitary regulation.

In the present review, the term *stress* is used operationally to describe physiological states characterized by one or more of the following conditions: negative energy balance, sustained physical workload, sleep restriction, or uncontrollable psychological challenge. These stressors engage integrated hypothalamic regulatory pathways that simultaneously influence hypothalamic–pituitary–adrenal and hypothalamic–pituitary–gonadal signaling. Clarifying the specific biological context of “stress” is essential because testosterone responses differ markedly depending on energetic status, duration of exposure, and perceived controllability (23).

### Early military evidence: Korea- and Vietnam-Era psychoendocrine field studies

Foundational military investigations conducted during the Korean and Vietnam wars established that severe operational stress does not produce uniform or global endocrine failure, but instead elicits selective, centrally mediated hormonal adaptations (24, 25). Early front-line endocrine studies from the Korean War, based on complete 24-hour urine collections obtained under combat conditions, demonstrated a striking dissociation between stress axes. Twenty-four–hour urinary 17-hydroxycorticosteroid (17-OHCS) excretion increased appropriately during acute artillery threat but did not exhibit progressive decline with prolonged front-line exposure (24, 25). Because 17-OHCS reflects total daily glucocorticoid secretion rather than transient serum peaks, these findings indicate preserved ACTH–adrenal responsiveness and regulated HPA activation rather than global endocrine failure.

Vietnam-era studies extended these observations by demonstrating that psychological stress responses were highly context- and role-dependent, rather than proportional to objective exposure to danger. Psychoendocrine field investigations of helicopter ambulance crews and Special Forces personnel showed that anxiety and perceived threat fluctuated with mission demands, appraisal, and perceived control, while endocrine responses varied accordingly (26-30). These findings reinforced the conclusion that stress physiology is governed by central appraisal mechanisms and that sustained exposure to danger may be accompanied by physiological stabilization rather than progressive endocrine breakdown (16, 31, 32). Importantly, these field observations predated and anticipated later laboratory and training-based evidence showing that psychogenic stress can selectively modulate gonadal function.

Direct evidence that psychological stress suppresses circulating testosterone in humans emerged from controlled military training environments. Testosterone concentrations were significantly reduced during the early, highly stressful phases of Officer Candidate School training compared with later phases characterized by adaptation and predictability (31). These findings established that psychogenic stress alone, independent of overt physical exhaustion or caloric deprivation, can suppress testosterone via central mechanisms, extending stress physiology beyond the HPA axis to include functional inhibition of the hypothalamic–pituitary–gonadal (HPG) axis.

### Energy deficiency is the dominant determinant of androgen suppression

Across military field studies spanning more than five decades, sustained energy deficiency, particularly when coupled with prolonged physical work and sleep restriction, emerges as the most potent and reproducible determinant of testosterone suppression under operational stress. Across these field studies, reductions in testosterone are accompanied by reduced LH pulse amplitude and/or frequency, while Leydig cell responsiveness to exogenous human chorionic gonadotropin remains intact. This pattern confirms diminished endogenous gonadotropin drive as the dominant mechanism rather than intrinsic testicular failure (22, 33-37).

The most compelling mechanistic evidence derives from the comprehensive field experiments conducted by Per Kristian Opstad and colleagues in Norwegian officer cadets undergoing multi-day endurance and survival courses (1, 8, 9, 38). These studies combined extreme energetic deficit (daily energy expenditures averaging 26.6 MJ/d with caloric intakes between 0.2 and 2.2 MJ/day), with near-continuous physical activity, and profound sleep loss (typically ≤1-3 hours total sleep over 4-6 days) (1, 9, 39). Under these conditions, plasma concentrations of total testosterone, free testosterone, dihydrotestosterone, androstenedione, and dehydroepiandrosterone declined rapidly and markedly, often by 60-80% within 72-120 hours, in parallel with reductions in luteinizing hormone (LH) and follicle-stimulating hormone (FSH) (9). This coordinated suppression implicates hypothalamic–pituitary inhibition as the primary regulatory mechanism.

Opstad's work directly addressed whether this androgen suppression reflected intrinsic testicular dysfunction. In carefully executed human chorionic gonadotropin (hCG) stimulation tests, testosterone concentrations normalized promptly following stimulation, confirming preserved Leydig cell responsiveness and arguing against intrinsic gonadal failure (1, 19). These findings demonstrate centrally mediated suppression of gonadotropin drive with preserved gonadal capacity. The failure of testosterone to recover under unstimulated conditions was attributed to central suppression and circadian extinction, not impaired steroidogenic capacity. These experiments provide rare field-based causal evidence that so-called “operational hypogonadism” is functional and centrally mediated, rather than pathological. This is analogous to elegant laboratory studies of energy expenditure and deficiency in women, where, independently of energy expenditure, increasing energy deficit produces a decrease in LH pulse frequency (20). Not all operational stress paradigms suppress gonadotropin secretion. In a controlled 84-hour military model combining sustained physical exertion, caloric restriction, and sleep deprivation, total testosterone declined by approximately 24% and free testosterone by approximately 30%, despite a 46% increase in luteinizing hormone (LH) pulse amplitude and preserved pulse frequency (40). This divergence indicates maintained, and potentially compensatory, pituitary drive in the setting of reduced testicular steroidogenic output, consistent with acute peripheral resistance rather than primary hypothalamic inhibition. These findings suggest that early or severe metabolic stress may initially provoke compensatory LH amplification before transition to centrally mediated suppression during more prolonged or energy-deficient states. These findings suggest a stage-dependent response, with early compensatory LH amplification preceding central suppression during prolonged stress.

**Figure 1:**
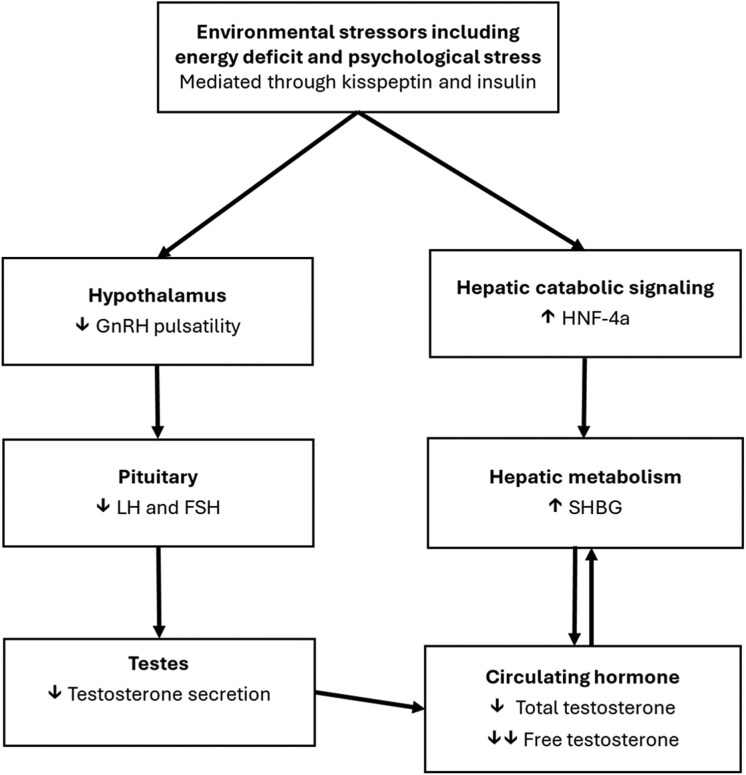
Coordinated central and hepatic adaptations underlying stress-associated reductions in testosterone. Operational stressors, including energy deficit and psychological stress, reduce hypothalamic GnRH pulsatility and downstream LH/FSH secretion, leading to reduced testicular testosterone production and secretion. In parallel, hepatic metabolic signaling increases sex hormone-binding globulin (SHBG) expression through pathways involving hepatocyte nuclear factor-4a (HNF-4a), which further lowers circulating free testosterone. These coordinated central and hepatic responses produce reductions in both total and free testosterone during sustained stress, reflecting a centrally mediated reprioritization of endocrine function rather than primary testicular failure.

A further mechanistic insight concerns hormone binding and androgen bioavailability. Prolonged energy deficit and stress exposure were accompanied by significant increases in SHBG, amplifying reductions in bioavailable testosterone despite preserved gonadal capacity (1, 8, 33). This dual mechanism of reduced gonadotropin-driven secretion combined with increased binding amplifies the suppression of biologically active androgen signaling at the tissue level (1, 8, 33). This represents a highly efficient means of down-regulating androgen-dependent anabolic processes during sustained energy scarcity, even when total testosterone concentrations remain within low-normal clinical ranges (Fig. 1). Opstad also observed a reduction in androgen action based on a reduction in beard growth in the cadets by the second day of energy restriction (9).

Nutritional countermeasures alone were insufficient to fully restore androgen concentrations, while severe physical strain and sleep deprivation persisted (33). Provision of high-calorie diets partially attenuated cortisol elevations and modified growth hormone responses but did not normalize testosterone until the broader stress ecology, including workload and sleep debt, was resolved. In a similar but more protracted energy restriction model, US Army Ranger students subjected to intermittent feeding averaging an energy deficit of 1000-1200 kcal/d for 8 weeks demonstrated the same ∼50% decrease in testosterone, declines in LH, and rise in SHBG that were observed in the compressed 1-week food restriction timeline in the Norwegian training (1, 2). During brief refeeding and recovery periods (eg, base-camp intervals and post-course recovery), testosterone concentrations recovered to baseline levels. Testosterone concentration (average of three samples during the course) had modest significant correlation (r = 0.3, n = 105 men) with preservation of fat-free mass; the greatest effect was initial body fat, where body energy stores delayed the consumption of muscle mass protein for energy needs (41).

Interpretation of testosterone concentrations in historical studies should also consider assay methodology. Earlier investigations frequently relied on immunoassays with limited analytical specificity at low concentrations, whereas modern studies increasingly employ liquid chromatography–tandem mass spectrometry (LC–MS/MS), which provides improved accuracy and reduced cross-reactivity (42). Some of the Ranger course studies have been repeated using modern techniques of LC-MS/MS and confirmed earlier findings (43).

The biological logic of this response is reinforced by parallel endocrine adaptations documented in these studies, including suppression of thyroid hormones, extinction of diurnal steroid rhythms, and coordinated shifts toward catabolic metabolism and energy mobilization (2, 39, 44). As noted by Opstad, several of these alterations may be advantageous or necessary during sustained military operations, becoming maladaptive only when prolonged beyond the operational context.

Closely analogous endocrine phenotypes are now well-documented in endurance athletes experiencing chronic low energy availability, formalized within the framework of relative energy deficiency in sport (12, 20, 22, 45). Across the spectrum of soldiers and athletes, energy deficiency, not physical workload, determines the depth and duration of testosterone suppression, whereas individuals exposed to high training volumes but maintaining adequate energy intake and sleep generally preserve normal free testosterone concentrations.

Taken together, the military field studies establish sustained energy deficiency as the dominant determinant of androgen suppression during training and operations, operating through centrally mediated suppression of gonadotropin secretion and amplified by increased SHBG binding (1, 2, 35, 37, 46-48). Recognition of this adaptive endocrine pattern is essential for the correct interpretation of testosterone measurements in military and athletic settings and for avoiding misclassification of reversible stress physiology as pathological hypogonadism.

### Psychogenic stress, social control, and androgen regulation

Psychogenic stress modulates androgen signaling through centrally mediated mechanisms that depend more on appraisal, perceived control, and social status than on physical stress exposure alone. Early military psychoendocrine investigations during the Korean and Vietnam wars demonstrated that physiological responses to threat were heterogeneous within units and closely linked to role demands and perceived agency rather than objective danger (24-29). These field observations anticipated later experimental models of dominance, competition, and social defeat and established a critical principle: activation of the hypothalamic–pituitary–gonadal (HPG) axis is contingent on context and control, not simply arousal.

The energetic costs of androgen signaling provide a biological basis for this context sensitivity. Testosterone has measurable metabolic costs, including elevation of basal metabolic rate and increased energetic investment in anabolic tissue and dominance signaling (10, 49). From an evolutionary perspective, reproductive and anabolic investment should be curtailed when environmental predictability, social status, or energetic security are threatened (11, 15). Experimental and cross-cultural human data demonstrate that testosterone-mediated functions are integrated within broader life-history trade-offs involving immunity, survival, and reproductive timing (14, 49).

Foundational evidence for this model emerged from socially housed male rhesus monkeys, in whom circulating testosterone concentrations were systematically related to dominance rank (50, 51). Dominant animals exhibited higher testosterone than subordinates despite comparable physical environments and activity levels. Importantly, testosterone tracked stability of rank and effective social control rather than frequency of aggressive behavior per se. Subordinate animals with high aggression, but low social control, often exhibited suppressed testosterone, indicating that agency and outcome predictability, not behavioral activation alone, determine androgen status. These findings informed subsequent biosocial models of status and dominance in humans (6, 52-54).

Parallel patterns were documented in military settings. In Vietnam-era Special Forces observations, leaders under threat sometimes exhibited psychophysiological responses distinct from those of the soldiers they commanded, despite identical exposure to danger (26, 27). Individuals embedded in decision-making roles frequently demonstrated stabilized endocrine patterns compared with subordinates whose roles involved vigilance and limited control. These findings reinforced the conclusion that stress endocrinology reflects cognitive appraisal and role-based controllability rather than stimulus intensity alone (11).

Direct human evidence that psychological stress suppresses testosterone independent of energy deficit was provided in longitudinal studies of Army Officer Candidate School trainees. During the early phase of training, characterized by continuous evaluation, rank ordering, and social-evaluative threat, plasma testosterone concentrations were significantly reduced compared with later phases marked by adaptation and increased predictability (31). More recent officer training studies similarly demonstrate divergence of testosterone and cortisol responses according to stress appraisal and chronicity (5). Contemporary dynamic testing in British Army Officer Cadets undergoing prolonged military training showed progressive HPA amplification and modulation of gonadotropin responsiveness without evidence of intrinsic gonadal failure (55). These changes were not fully explained by energy balance, reinforcing that sustained operational and psychogenic stress modulate testosterone through coordinated centrally mediated suppression of gonadotropin drive rather than intrinsic gonadal failure. Collectively, these findings extend psychoendocrine models beyond the HPA axis and confirm that psychological stress can centrally suppress gonadotropin drive.

Theoretical integration of these observations was articulated within coping-based stress models emphasizing controllability and expectancy. In Norwegian paratrooper training, initial high-threat exposures elicited suppression of testosterone and elevation of cortisol, whereas repeated successful exposures produced transient post-event elevations in testosterone with normalization of cortisol, consistent with active coping and mastery (16). Similar outcome-dependent responses are observed in athletic competition. Victory or perceived dominance is associated with transient increases or preservation of testosterone, whereas defeat or loss of control produces suppression, even when physical exertion is equivalent (56-60). Cortisol responses frequently rise in both winners and losers, underscoring that HPA activation and HPG suppression are not obligatorily coupled (57, 58). These rapid increases in testosterone during acute psychological challenge do not contradict the adaptive suppression model; rather, they illustrate the dynamic central regulation of the hypothalamic–pituitary–gonadal axis, which can transiently enhance or suppress androgen output depending on energetic context, controllability, and perceived challenge.

Testosterone regulation under psychogenic stress must be interpreted within broader energetic and immunologic trade-offs (14, 49). When stress is appraised as uncontrollable or defeat-like, suppression of androgen signaling may conserve energy and preserve host defense capacity (11, 14). This interpretation aligns with evidence from military multi-stressor environments in which androgen suppression co-occurs with immune perturbations and negative energy balance (2, 47, 61-63).

### Athlete studies: HPG regulation under exercise, energy constraint, and competitive appraisal

Athlete populations provide a controlled physiological model for examining the HPG axis regulation under conditions in which physical workload, energy availability, sleep, and psychogenic stress can be partially dissociated. Across endurance and resistance training paradigms, basal testosterone concentrations are not determined by exercise modality per se, but by the energetic and neuroendocrine context in which training occurs (2, 13, 64).

In endurance-trained men, particularly those exposed to sustained high training volumes with marginal energy availability, resting total testosterone concentrations frequently reside in the low–normal range; SHBG elevation is common in endurance cohorts, resulting in disproportionate reductions in calculated free testosterone (3, 8, 17, 65). This phenotype, termed the “exercise hypogonadal male condition,” has been documented in distance runners and other high-volume aerobic athletes and is characterized by reduced luteinizing hormone pulsatility and preserved testicular responsiveness, implicating central suppression rather than intrinsic gonadal failure (3). The magnitude of suppression correlates more strongly with indices of energy availability than with aerobic workload itself, and restoration of caloric sufficiency and sleep regularity normalizes testosterone and SHBG concentrations, supporting a reversible, centrally mediated mechanism (4, 65).

Comparable endocrine patterns are observed in prolonged military field exercises characterized by negative energy balance and sleep restriction, where reductions in testosterone are accompanied by alterations in cortisol dynamics and immune indices (2, 4, 48). These convergent findings reinforce that sustained energy constraint, rather than mechanical loading or cardiorespiratory stress alone, is the dominant regulator of basal androgen output.

In contrast, resistance-trained men who maintain adequate long-term energy availability and recovery do not exhibit sustained suppression of basal testosterone despite repeated exposure to high mechanical and metabolic stress (12, 13, 66). Acute resistance exercise induces transient elevations in circulating testosterone, particularly when large muscle mass is engaged, reflecting short-term hypothalamic and pituitary activation (66-68). However, high-volume resistance exercise can also produce short-lived reductions in overnight total and free testosterone, accompanied by a blunted LH production rate, consistent with centrally mediated suppression of gonadotropin drive rather than intrinsic testicular dysfunction (18). These effects appear to reflect transient energetic and neuroendocrine strain rather than a sustained downregulation of the HPG axis. Over time, chronic resistance training in eugonadal men does not reliably elevate basal testosterone nor does it produce persistent suppression when energy balance and recovery are maintained (2, 13, 64, 67). Together, these findings suggest that tissue loading alone is insufficient to chronically downregulate the HPG axis and that energetic sufficiency and cumulative stress exposure modulate basal androgen tone more strongly than exercise modality itself. Acute competitive stress provides a useful contrast to sustained stress models. In short-duration, controllable, appraisal-dependent contexts, central neural input to the HPG axis can transiently augment testosterone secretion without altering SHBG, consistent with preserved or stimulated LH drive (56-59). These increases are typically brief, resolving within hours. When stress becomes prolonged, energetically constraining, or perceived as uncontrollable, LH pulsatility declines and testosterone suppression emerges. Early activation and later suppression therefore represent temporally distinct manifestations of central regulation.

Across combat sports and other competitive settings, testosterone responses diverge according to outcome appraisal: victory and perceived dominance are associated with transient increases or preservation of testosterone, whereas defeat and loss of control produce acute suppression (56-59). These responses occur rapidly, are centrally mediated, and typically occur without meaningful changes in SHBG, distinguishing acute psychogenic modulation from the binding-mediated reductions observed during prolonged energy deficiency (56-59). Cortisol frequently rises in both winners and losers, underscoring that hypothalamic–pituitary–adrenal activation and HPG suppression are not obligatorily coupled (57, 58).

Across athletic and military contexts, chronic reductions in testosterone are most consistently explained by negative energy balance and sleep disruption, whereas exercise modality alone exerts modest and often transient effects. Acute fluctuations in testosterone track appraisal and central drive more closely than peripheral tissue damage. These data reinforce the central thesis of this manuscript: testosterone regulation reflects coordinated hypothalamic reprioritization in response to energetic and psychological context rather than intrinsic gonadal failure.

### Clinical and readiness implications

**Figure 2:**
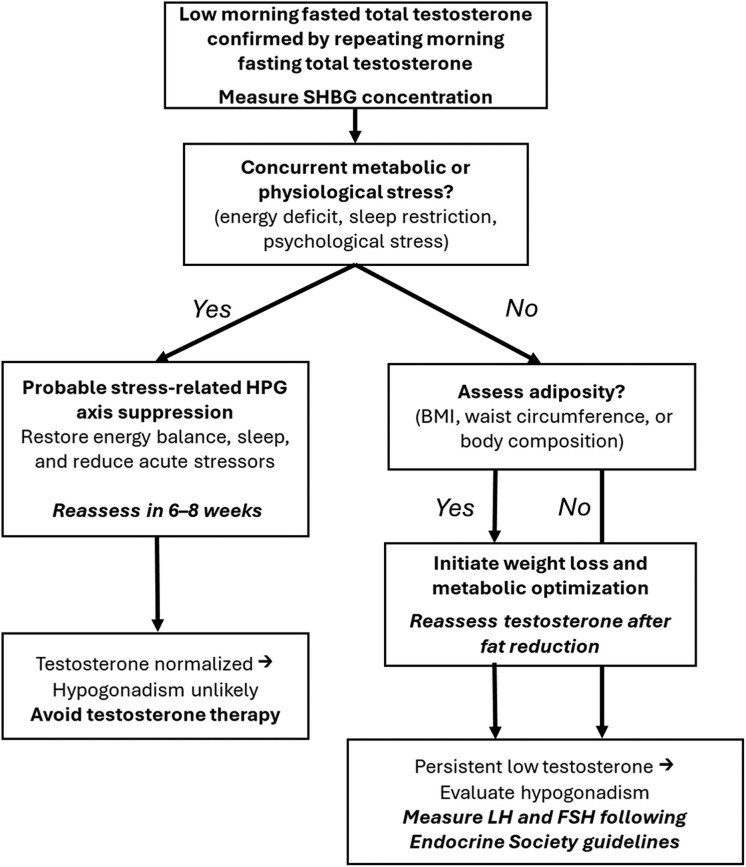
Physiological suppression of testosterone during systemic stress, distinguishing adaptive endocrine responses from hypogonadism. Clinical decision framework for interpreting low morning testosterone measured during metabolic or operational stress. Low values should first be confirmed with repeat morning fasting testosterone and assessment of sex hormone-binding globulin (SHBG). Elevated SHBG and transient suppression during energy deficit, sleep restriction, or psychological stress suggest adaptive hypothalamic-pituitary-gonadal axis downregulation. Restoration of energy balance and sleep with reassessment is recommended before considering testosterone therapy. Persistent biochemical hypogonadism warrants evaluation with LH and FSH according to endocrine clinical practice guidelines.

Most field-related suppression of testosterone in athletes and soldiers is functional and reversible. This normal physiological regulation is self-correcting with sleep, energy intake, and reduction or removal of the stress load. In these conditions, testosterone is a barometer, not a treatment target, and failure to recognize adaptive testosterone suppression risks unnecessary diagnostic labeling and inappropriate intervention. Intervention may even be harmful, such as forcing anabolism during energy deficiency at the cost of other functions, such as immune defenses. Accordingly, clinical interpretation of low testosterone in physically active or operational populations requires systematic evaluation of energy availability, sleep integrity, psychogenic stress exposure, and body composition before considering intrinsic hypogonadism, an approach formalized in the decision framework presented in Fig. 2.

Severe multistressor conditions characterized by sustained energy deficit, sleep deprivation, and prolonged physical strain have important implications for host defense. Controlled military field studies demonstrate that such conditions can impair immune function, including reductions in lymphocyte proliferation and altered cytokine signaling (61, 62) and increase susceptibility to infection during prolonged training exercises in field environments where pathogen exposure is common (47, 63, 69). These immune changes occur in parallel with the endocrine adaptations described above and reflect coordinated physiological prioritization under conditions of energetic constraint. While experimental models of extreme stress conducted in controlled laboratory environments provide valuable mechanistic insight, the same exposures in operational training environments, where pathogen exposure is unavoidable, may carry greater medical risk (70). Accordingly, while controlled studies of severe stress physiology are academically informative, extrapolation to operational training environments must be undertaken cautiously to avoid compromising health or readiness. Concerns are further heightened by sporadic reports of severe illness during extreme military training environments when individuals simultaneously employ performance-enhancing substances, including androgenic-anabolic steroids. Although causal relationships are difficult to establish, the combination of immunologic strain, sleep deprivation, and pharmacologic androgen exposure may increase medical risk under extreme operational conditions.

Accurate interpretation of testosterone in healthy active men requires attention to binding, bioavailability, and stress context rather than reliance on total testosterone alone. Many research studies report only a single morning testosterone concentration, and this needs to be interpreted considering the context. For example, acute shifts in the vascular fluid volume (eg, following intense exercise) will produce hemoconcentration of the bound testosterone and the appearance of an elevated testosterone (71).

Modern research investigations are increasingly using LC-MS/MS, which provides improved analytical accuracy and reduced cross-reactivity (72). Methodological concerns still apply to salivary testosterone measurements commonly used in behavioral studies, where micro-contamination with blood may introduce substantial analytical variability. Consequently, studies relying on salivary immunoassay measurements should be interpreted cautiously when drawing conclusions about physiological testosterone regulation. Standardization of testosterone laboratory protocols (eg, CDC Hormones Standardization Programs) improve accuracy and reduce variability, with a goal to reduce precision to < 5.3% and bias to less than ± 6.4% for a total error of < 16.7% (73). However, day-to-day biological variability is still high, and the difference between two samples collected on separate days from the same individual could be as high as 50% for some of the men (71). Batch assays are an important consideration in longitudinal studies to avoid intra-assay error or assay drift errors. Calculated free testosterone is widely used in clinical interpretation but depends on assumptions regarding binding equilibria and SHBG concentrations, and its physiological interpretation remains debated (74). Sleep disruption or restriction may abolish circadian rhythms, producing apparently low morning testosterone concentrations, as demonstrated by Opstad within the first day of training in the Norwegian cadet studies (44). In studies with young men, supraphysiological supplementation with an anabolic-androgenic steroid such as nandrolone or an alkylated testosterone such as methyl testosterone produces a pseudo-hypogonadism with suppressed endogenous testosterone secretion and abolished diurnal rhythms (75, 76).

Many healthy young men are under the common misconception that they need higher testosterone, based on rampant misinformation on the internet. Without question, there is widespread overprescription of testosterone therapy (77). For individuals who are persistently in the hypogonadal range of testosterone, an initial consideration should be the factors discussed in this paper, followed by assessment of fitness and obesity. Excess adiposity represents a chronic energetic state with androgen metabolism altered through increased aromatization and inflammatory signaling, and specific metabolic disruption associated with high visceral adipose tissue (78). Although visceral fat is androgen-sensitive, testosterone treatment has only modest effects on adiposity and visceral fat (79). In this context, reduced circulating testosterone reflects both metabolic regulation and altered hormone binding rather than intrinsic gonadal failure. Weight reduction frequently restores testosterone concentrations, reinforcing the importance of metabolic context when interpreting low testosterone (7).

## Summary and conclusions

Testosterone suppression during sustained stress states is neither uniform nor inherently pathological. Convergent evidence from prolonged military operations, controlled energy-deficit studies, athletic models, and competitive stress paradigms demonstrates that reduced circulating testosterone commonly reflects centrally mediated suppression of gonadotropin drive rather than intrinsic testicular failure. Under conditions of negative energy balance, sleep restriction, and uncontrollable psychogenic stress, hypothalamic reprioritization suppresses luteinizing hormone pulsatility, reduces testicular androgen output, and frequently increases sex hormone–binding globulin, thereby lowering free testosterone. Human stimulation studies confirm preserved Leydig cell responsiveness, establishing that reduced circulating concentrations arise primarily from diminished endogenous gonadotropin stimulation.

Testosterone regulation during stress is temporally dynamic. Short-duration, controllable challenges may transiently increase androgen output through central activation. In contrast, sustained energetic deficit, sleep disruption, and uncontrollable stress suppress LH pulsatility, reduce endogenous testicular stimulation, and lower circulating testosterone despite intact gonadal capacity. Recognition of this trajectory distinguishes adaptive central regulation from pathological hypogonadism and frames androgen suppression within the logic of energy allocation. Primate studies of pulsatile GnRH signaling established that suppression of testosterone during sustained stress is best understood as centrally mediated alteration of hypothalamic pulse generation and LH drive rather than evidence of intrinsic gonadal dysfunction.

This adaptive endocrine phenotype is reversible with restoration of energy availability, recovery, and reduction of sustained stressors. Persistent suppression in the absence of energetic or psychogenic constraint, however, warrants evaluation for intrinsic hypogonadal pathology. Distinguishing functional central suppression from intrinsic hypogonadism aligns clinical evaluation with the adaptive logic of human stress physiology. Randomized trials of testosterone therapy have demonstrated modest improvements in mood and depressive symptoms in men with low testosterone concentrations. However, these studies largely involve aging populations or men with chronic depressive disorders rather than individuals experiencing reversible stress-associated suppression. Thus, while testosterone therapy may improve mood in selected clinical contexts, it does not address the underlying physiological drivers of stress-related androgen suppression, such as energy deficit, sleep deprivation, or psychological strain. Testosterone replacement therapy is not a substitute for adequate nutrition, recovery, or psychological stability, and attempts to pharmacologically override adaptive endocrine suppression during sustained stress may carry physiological risks without correcting the underlying drivers of suppression.

## Data Availability

Data sharing is not applicable to this article as no datasets were generated or analyzed during the current study.
